# Galactose 6-*O*-Sulfotransferases Are Not Required for the Generation of Siglec-F Ligands in Leukocytes or Lung Tissue*

**DOI:** 10.1074/jbc.M113.485409

**Published:** 2013-07-23

**Authors:** Michael L. Patnode, Chu-Wen Cheng, Chi-Chi Chou, Mark S. Singer, Matilda S. Elin, Kenji Uchimura, Paul R. Crocker, Kay-Hooi Khoo, Steven D. Rosen

**Affiliations:** From the ‡ Department of Anatomy and Program in Biomedical Sciences, University of California, San Francisco, California 94143-0452,; the § Institute of Biological Chemistry, Academia Sinica, Taipei 11529, Taiwan,; the ‖ Division of Cell Signaling and Immunology, College of Life Sciences, University of Dundee, Dundee DD1 5EH, Scotland, United Kingdom, and; the ¶ Department of Biochemistry, Nagoya University Graduate School of Medicine, Aichi 466-8550, Japan

**Keywords:** Eosinophils, Lectin, Lung, Mucins, Sulfotransferase, Galactose-6-O-Sulfate, Siglec-F

## Abstract

Eosinophil accumulation is a characteristic feature of the immune response to parasitic worms and allergens. The cell surface carbohydrate-binding receptor Siglec-F is highly expressed on eosinophils and negatively regulates their accumulation during inflammation. Although endogenous ligands for Siglec-F have yet to be biochemically defined, binding studies using glycan arrays have implicated galactose 6-*O*-sulfate (Gal6S) as a partial recognition determinant for this receptor. Only two sulfotransferases are known to generate Gal6S, namely keratan sulfate galactose 6-*O*-sulfotransferase (KSGal6ST) and chondroitin 6-*O*-sulfotransferase 1 (C6ST-1). Here we use mice deficient in both KSGal6ST and C6ST-1 to determine whether these sulfotransferases are required for the generation of endogenous Siglec-F ligands. First, we characterize ligand expression on leukocyte populations and find that ligands are predominantly expressed on cell types also expressing Siglec-F, namely eosinophils, neutrophils, and alveolar macrophages. We also detect Siglec-F ligand activity in bronchoalveolar lavage fluid fractions containing polymeric secreted mucins, including MUC5B. Consistent with these observations, ligands in the lung increase dramatically during infection with the parasitic nematode, *Nippostrongylus brasiliensis*, which is known to induce eosinophil accumulation and mucus production. Surprisingly, Gal6S is undetectable in sialylated glycans from eosinophils and BAL fluid analyzed by mass spectrometry. Furthermore, none of the ligands we describe are diminished in mice lacking KSGal6ST and C6ST-1, indicating that neither of the known galactose 6-*O*-sulfotransferases is required for ligand synthesis. These results establish that ligands for Siglec-F are present on several cell types that are relevant during allergic lung inflammation and argue against the widely held view that Gal6S is critical for glycan recognition by this receptor.

## Teaser

**Background:** The cell surface lectin Siglec-F is thought to preferentially recognize ligands modified with galactose 6-*O*-sulfate.

**Results:** Siglec-F ligands are still present in leukocytes and lung tissue from mice lacking galactose 6-*O*-sulfotransferases.

**Conclusion:** Ligands are restricted to specific cell types, but galactose 6-*O*-sulfotransferases are not required for ligand binding.

**Significance:** This study refines our understanding of the biological ligands for Siglec-F.

## Introduction

Eosinophils are circulating leukocytes that are normally rare in blood and tissues. However, these cells characteristically accumulate during immune responses against multicellular parasites (1). Eosinophils surround helminths in host tissues, and significantly reduce parasite load in several animal models of infection. Additionally, eosinophil accumulation is associated with allergic disease. In mouse models of asthma (2) and atopic dermatitis (3), eosinophils promote tissue remodeling and fibrosis. Eosinophil activation is mediated by cell surface receptors including cytokine receptors, Fc receptors, integrins, and C-type lectins (1). Engagement of these receptors triggers the release of a multitude of cytokines, eicosanoids, and granule proteins. Because these products can have detrimental effects on host physiology, eosinophil activation is likely to be restricted by inhibitory cell surface receptors similar to those that dampen lymphocyte and NK cell activation (4). Although no receptor has conclusively been demonstrated to serve this function *in vivo*, several putative inhibitory receptors have been identified on eosinophils (5–7). Prominent among them is Siglec-F, the subject of the present study.

Sialic acid-binding immunoglobulin-like lectins (Siglecs)^2^ are a family of cell surface carbohydrate-binding receptors primarily expressed on circulating and tissue-resident leukocytes (8, 9). These receptors are comprised of a variable number of extracellular C2-set immunoglobulin (Ig) domains and an N-terminal V-set Ig domain with carbohydrate binding activity. The cytoplasmic domains of most Siglecs contain sequences resembling immunoreceptor tyrosine-based inhibition motifs, which can recruit inhibitory phosphatases to the signaling complexes generated by activating receptors. All Siglecs recognize glycans that terminate in sialic acid, but each has a distinct binding profile with preferences for the linkage of sialic acid together with features of the underlying carbohydrate structure. Siglecs expressed by a given cell can bind glycoproteins and glycolipids on the surface of that same cell (referred to as cis-ligands) or on another cell (referred to as trans-ligands). Siglecs can also bind secreted ligands, such as mucins (10–12). Four members of the Siglec family, sialoadhesin, CD22, MAG, and Siglec-15, are well conserved among mammals. In contrast, the CD33-related Siglecs are rapidly evolving, and there are no clear orthologs between mice and humans, with the exception of Siglec-G and Siglec-10. Thus, the CD33-related Siglecs have been assigned the letters E through H in mice and the numbers 5 through 14 in humans. The strikingly restricted expression patterns of several Siglecs such as sialoadhesin (Siglec-1, CD169) on subsets of macrophages (13), Siglec-H on plasmacytoid dendritic cells (14), and Siglec-F on eosinophils (15) suggests that they carry out specialized functions, but in most cases these functions are incompletely understood.

Siglec-F is expressed by eosinophil precursors in the bone marrow and is constitutively present on mature eosinophils (15, 16). The level of Siglec-F on eosinophils also increases during allergic inflammation and upon their migration into tissues (16, 17). Apart from eosinophils, only alveolar macrophages prominently express Siglec-F (18), although weak expression has been detected on neutrophils and on T cells during *in vitro* activation (17). Although the consequences of ligand recognition by Siglec-F are still unclear, this receptor has been shown to promote apoptosis in eosinophils upon antibody cross-linking (17, 19). Additionally, intravenous injection of either intact antibodies or F(ab′) fragments directed against Siglec-F markedly depletes eosinophils from blood and tissues (19–21). In agreement with these findings, Siglec-F KO mice show systemic increases in eosinophil numbers during ovalbumin-induced lung inflammation, and a decrease in the number of apoptotic cells in the lung (17, 22). Thus, it has been proposed that ligands for Siglec-F may be important for dampening eosinophil accumulation during inflammation (23).

Potential endogenous ligands for Siglec-F can be detected indirectly through the use of sialylated glycans linked to polyacrylamide scaffolds (24). Eosinophils do not bind these glycans unless the eosinophils are first treated with sialidase, suggesting that the ligand-binding site of Siglec-F is occupied by cis-ligands, as is the case for CD22 and several other Siglecs (9). Direct detection of Siglec-F ligands in lung tissue has been achieved using a fusion protein consisting of the extracellular domain of Siglec-F fused to the Fc portion of human IgG (Siglec-F-Fc). Immunohistochemical staining of mouse lung sections with Siglec-F-Fc reveals sialic acid-dependent ligands on airway epithelial cells and luminal contents, as well as on mononuclear cells in alveolar spaces (17). Furthermore, Siglec-F-Fc staining in these regions increases dramatically during allergic lung inflammation. However, the identities of these ligand expressing cells and the basis for the increase in ligands during inflammation have not been thoroughly investigated.

Although endogenous ligands have yet to be biochemically defined, experiments using polyacrylamide-linked glycans have established that Siglec-F prefers α2,3-linked sialic acid residues (25). Consistent with this specificity, Siglec-F-Fc staining of airway epithelium and alveolar cells is blocked by *Maackia amurensis* agglutinin which recognizes α2,3-linked sialic acids, and absent in mice lacking the α2,3-sialyltransferase ST3Gal3 (26, 27). Additionally, Siglec-F-Fc specificity has been probed using the Consortium for Functional Glycomics glycan array, which consists of several hundred different structures (28). These experiments reveal a striking preference for 6′-sulfo-sLex (Siaα2→3(6S)Galβ1→4(Fucα1→3)GlcNAc) and 6′-sulfo-3′sLN (Siaα2→3(6S)Galβ1→4GlcNAc) (24), consistent with a requirement for α2,3-linked sialic acid. Importantly, these data also demonstrate a requirement for a sulfate modification on the 6-*O* position of galactose. Siglec-F-Fc binds neoglycolipids modified with 6′-sulfo-sLex, 6′-sulfo-3′sLN, and 6,6′-sulfo-sLex (Siaα2→3(6S)Galβ1→4(Fucα1→3)(6S)GlcNAc), but not sLex (Siaα2→3Galβ1→4(Fucα1→3)GlcNAc) or 6-sulfo-sLex (Siaα2→3Galβ1→4(Fucα1→3)(6S)GlcNAc) (29). 6′-Sulfo-sLex, but not 6-sulfo-sLex, coupled to polyacrylamide binds to de-sialylated eosinophils in a Siglec-F dependent manner (24). Additionally, an antibody that recognizes 6′-sulfo-sLex stains mouse airway epithelium where Siglec-F ligands have been detected (30), although this antibody also binds other glycan structures. Based on these data, the prevailing view has been that galactose 6-*O*-sulfate (Gal6S) is likely to be a critical recognition element for Siglec-F *in vivo*.

Galactose 6-*O*-sulfotransferases (Gal6STs) catalyze the transfer of sulfate from the universal donor 3′-phosphoadenosine 5′-phosphosulfate (PAPS) to the 6-*O* position of Gal. The only sulfotransferases in mammals that are known to generate Gal6S are keratan sulfate galactose 6-*O*-sulfotransferase (KSGal6ST, encoded by the gene *Chst1*) and chondroitin 6-*O*-sulfotransferase-1 (C6ST-1, encoded by the gene *Chst3*). These enzymes belong to the GlcNAc/Gal/GalNAc-6-*O-*sulfotransferase (GST) subfamily. The four other members of this subfamily are known to possess GlcNAc 6-*O*-sulfotransferase activity (31). Biochemical studies have established that KSGal6ST and C6ST-1 can catalyze the addition of sulfate to the 6-*O* position of Gal on extended keratan sulfate (KS) chains, which are comprised of repeating Galβ1→4(6S)GlcNAc units (32, 33). Importantly, both sulfotransferases can also add sulfate to smaller, sialylated oligosaccharides such as those recognized by Siglec-F on glycan arrays (34, 35). We recently demonstrated that KSGal6ST generates Gal6S *in vivo* on ocular KS, and on glycans in lymph nodes, including 6,6′-disulfo-3′sLN in high endothelial venules (36). Furthermore, KSGal6ST is expressed in the lung (37) and has recently been detected in airway epithelium by immunohistochemistry (26). Neither KSGal6ST nor C6ST-1 has been previously examined with respect to its ability to generate Siglec-F ligands.

Although the human genome does not contain an ortholog of Siglec-F, another CD33-related Siglec, Siglec-8, is believed to be a functional paralog based on several common features (23). First, Siglec-8 is highly expressed on human eosinophils, although, unlike Siglec-F, it is found on basophils and mast cells but not on neutrophils. Second, antibody-mediated cross-linking of Siglec-8 induces eosinophil apoptosis (38), an effect that also occurs when polyvalent glycan ligands are used (39). Third, and most notable, Siglec-8 recognizes glycans containing Gal6S, such as 6′sulfo-sLex, 6,6′-disulfo-sLex, and 6′-sulfo-3′sLN, with even greater selectivity than Siglec-F (29, 40, 41). Although ligands for Siglec-8 and the cell types expressing them have not been identified, such studies could potentially be guided by the characterization of ligands for Siglec-F in mice.

Here, we report the direct detection of Siglec-F ligands on mouse eosinophils, neutrophils, and alveolar macrophages, all of which express Siglec-F. We also demonstrate the presence of ligands in type II alveolar epithelial cells (AECs) and in polymeric mucin-containing fractions of bronchoalveolar lavage (BAL) fluid. However, contrary to expectations, neither KSGal6ST nor C6ST-1 is required for the generation of these ligands.

## EXPERIMENTAL PROCEDURES

### 

#### 

##### Mice

Mice deficient in KSGal6ST (36), C6ST-1 (42), and Siglec-F (17), as well as 4get mice (43) and IL-5 transgenic mice (44) have been described previously. KSGal6ST/C6ST-1 double knock-out (DKO) mice were genotyped by PCR as described previously (36). Additionally, these mice failed to generate a Gal6S-dependent epitope (36) in lymph node high endothelial venules. Siglec-F, 4get, and IL-5 transgenic mice were genotyped by FACS of peripheral blood leukocytes stained with 1 μg/ml of PE rat anti-mouse Siglec-F (BD Pharmingen). C57BL/6J mice were obtained from Jackson Laboratories. All procedures involving animals were approved by the University California San Francisco Institutional Animal Care and Use Committee, and carried out in accordance with the guidelines established by the National Institutes of Health.

##### Flow Cytometry

Blood from wild type or IL-5 transgenic mice was collected through the right ventricle into 5 mm EDTA in PBS, and then treated with ammonium chloride buffer (150 mm NH_4_Cl, 10 mm KHCO_3_, 100 μm EDTA) to lyze erythrocytes. Alveolar macrophages were collected by BAL with PBS. Peritoneal cells were collected by peritoneal lavage 72 h after intraperitoneal injection of Brewer-modified thioglycollate medium (BD Bioscience). Cells were then incubated in Hanks' balanced salt solution at 37 °C for 1 h with 50 milliunits/ml of *Arthrobacter ureafaciens* sialidase (Roche Applied Sciences), which hydrolyzes α2,3- α2,6-, and α2,8-linkages. *Vibrio cholerae* sialidase (Roche Applied Sciences) at 50 milliunits/ml in Hanks' balanced salt solution also eliminated Siglec-F-Fc and Siglec-E-Fc staining, but not CD22-Fc staining, consistent with its preference for α2,3-linkages (45). Sialidases were screened by the manufacturer for the absence of protease activity. As expected, sialidase treatment did not affect the percentage of viable cells or the percentages or intensities of cells stained with any of the antibodies we used. Cells were incubated with 10 μg/ml of anti-mouse CD16/32 (clone 93, eBioscience) to block Fc receptors. To measure Siglec-F expression, cells were incubated with 1 μg/ml of PE rat anti-mouse Siglec-F. Individual Siglec-Fc proteins (46) or human IgG (Invitrogen) was incubated at 1.5 μg/ml with biotin goat anti-human IgG at 0.75 μg/ml and APC streptavidin at 0.375 μg/ml for 1 h, then added to cells on ice for 1 h. Cells from 4get mice were stained with an anti-mouse antibody mixture containing 1 μg/ml of PE Ly-6G (1A8), PerCP-Cy5.5 Ly-6C (HK1.4, eBioscience), PE-Cy7 CD3ϵ (17A2, Biolegend), APC-eFluor780 CD11b (M1/70, eBioscience), or a separate antibody mixture containing 1 μg/ml of PE NK1.1 (PK136), PerCP-Cy5.5 CD19 (1D3), PE-Cy7 CD49b (DX5), and APC-eFluor780 CD3ϵ (17A2, eBioscience) for 30 min. For experiments with WT and C6ST-1/KSGal6ST DKO blood, cells were stained with an antibody mixture containing 1 μg/ml of FITC Ly-6G (1A8), PE NK1.1, PerCP-Cy5.5 Ly-6C, PE-Cy7 F4/80 (BM8, Biolegend), and APC-eFluor780 CD11b with eosinophils identified as CD11b^+^, NK1.1^−^, Ly-6C^+^, Ly-6G^low^, F4/80^+^, and SSC^high^. All antibodies were from BD Pharmingen unless otherwise stated. Viability was determined by adding 50 μl/ml of 7-aminoactinomycin D solution (BD Pharmingen) or 0.5 μg/ml of DAPI (Invitrogen). Cells were analyzed using a FACSort cytometer equipped with CellQuest software or an LSRII equipped with FACSDiva software. All cytometers and acquisition software were manufactured by BD Biosciences. Further analysis was carried out using FlowJo software (Treestar Inc.). Mean fluorescence intensity (MFI) values were calculated as the geometric mean, adjusted by subtraction of the MFI for an identical population stained with isotype control antibody. Flow cytometry histograms are expressed as a percentage of the maximum events.

##### Immunofluorescence Microscopy

The left lobe of the lung was perfused with PBS, collected, and fixed in 2% paraformaldehyde in PBS for 1 h, then incubated in 30% sucrose overnight at 4 °C. Lungs were inflated with 50% OCT compound (Sakura Finetek) in PBS and embedded in OCT compound then frozen by immersion in 2-methylbutane chilled in liquid nitrogen. Tissues were sectioned at 5 μm thickness in a cryostat and then fixed in −20 °C acetone for 10 min. For sialidase experiments, sections were treated with 50 milliunits/ml of *A. ureafaciens* sialidase (Roche Applied Sciences) in Hanks' balanced salt solution buffer at 37 °C for 1 h. Sections were blocked for 1 h with 5% normal sera from mouse and goat (Sigma) diluted in 0.5% casein solution (PerkinElmer Life Sciences) according to the manufacturer's instructions. Antibodies against sialoadhesin (clone SER4, BioLegend), proSP-C (Millipore), eMBP (47), and CD11b (clone M1/70, eBioscience) were used at 1 μg/ml for 1 h. Nonspecific rat IgG_1_ (eBioscience) was used to establish background fluorescence. Individual Siglec-Fc proteins or human IgG was incubated with sections at 1 μg/ml for 24 h. Secondary reagents, biotin goat anti-rat light chain, Cy3 goat anti-rabbit IgG, biotin goat anti-human IgG, and APC streptavidin were obtained from Jackson ImmunoResearch and incubated with sections at 1 μg/ml for 30 min. For sialoadhesin and eMBP staining, HRP streptavidin and FITC tyramide (PerkinElmer Life Sciences) were used according to the manufacturer's instructions. All antibodies were diluted in 0.5% casein solution. All sections were incubated with DAPI at 0.5 μg/ml in PBS for 5 min to label nuclei. Images were acquired using a Nikon Optiphot microscope equipped with an AxioCam HR at fixed exposure or a Carl Zeiss Axio-Imager. Multi-channel images were created and processed in parallel using Photoshop software (Adobe).

##### BAL Fluid Fractionation and ELISA

BAL fluid fractionation was carried out as described previously (48) with minor modifications. Briefly, BAL was performed using water, and BAL fluid from 5 mice was pooled and lyophilized. Samples were resuspended in 0.5 ml of 4 m guanidine chloride and spun at 20,000 × *g* for 30 min to remove debris. A 18 × 1-cm column of Sepharose CL-2B (Sigma) was equilibrated with 4 m guanidine chloride and calibrated using blue dextran. BAL fluid samples were applied to the column and a model 2110 fraction collector (Bio-Rad) was calibrated to collect 1-ml fractions. The absorbance of each fraction was measured at 280 nm using a SmartSpec 3000 (Bio-Rad). For ELISAs, 12-μl aliquots of fractions were coated onto Immulon-2HB plates overnight. Wells were incubated in 50 mm acetate buffer, pH 5.5, at 37 °C for 1 h with or without 50 milliunits/ml of *A. ureafaciens* sialidase, then blocked with 3% BSA for 1 h. Individual Siglec-Fc proteins or human IgG was incubated at 2 μg/ml with biotin goat anti-human IgG at 1 μg/ml and AP-streptavidin at 0.5 μg/ml for 1 h, then added to wells for 1 h. Plates were washed and developed with *p*-nitrophenyl phosphate in 10% diethanolamine, pH 9.8, with 0.5 mm MgCl_2_. Optical density at 405 nm was measured using a model 680 microplate reader (Bio-Rad).

##### Parasitic Worm Infection

Parasite infections were carried out as previously described (49). Briefly, mice were anesthetized using isofluorane and injected subcutaneously at the base of the tail with 500 *Nippostrongylus brasiliensis* L3 larvae. Mice were maintained on water containing 2 g/liter of neomycin sulfate, 100 mg/liter of polymixin B for 5 days, and sacrificed after 9 days.

##### Mass Spectrometry

Permethylated non-sulfated and sulfated glycan samples were prepared from 120 × 10^6^ peripheral blood leukocytes (81% eosinophils, 9% neutrophils) from IL-5 transgenic mice, or from BAL fluid, and initially profiled by MALDI-MS analyses, as described previously (36, 50). Subsequent nanoLC-MS/MS analyses of the permethylated, sulfated glycans were performed on a nanoACQUITY UPLC System (Waters) coupled to an LTQ-Orbitrap Velos hybrid mass spectrometer (Thermo Scientific). The sample was dissolved in 5% acetonitrile containing 0.1% formic acid, loaded onto a 75-μm × 250-mm nanoACQUITY UPLC BEH130 column (Waters, Milford MA), and eluted at a constant flow rate of 300 nl/min, with a linear gradient of 10–70% acetonitrile (in 0.1% formic acid) in 22 min, followed by a sharp increase to 95% acetonitrile in 17 min and then held isocratically for another 10 min. For each data-dependent acquisition cycle, the full-scan MS spectrum (*m*/*z* 900–2000) was acquired in the Orbitrap at 60,000 resolution (at *m*/*z* 400) with automatic gain control target value of 5 × 10^6^. A target precursor inclusion list was applied to precede further selection of five of the most intense ions with a intensity threshold of 500 counts for collision-induced dissociation (CID) and 1000 counts for higher energy C-trap dissociation (HCD). The automatic gain control target value and normalized collision energy applied for CID and HCD experiments were set as 5,000, 50%, and 50,000, 100%, respectively. All MS/MS data were interpreted manually.

##### Preparation and Structural Analysis of Keratan Sulfate

Lung and eosinophil keratan sulfate was isolated and analyzed as described previously for heparan sulfate (51) with slight modifications. Lung left lobes were dissected out from three adult mice (∼150 mg), and peripheral blood leukocytes (60 × 10^6^) were obtained from the blood of IL-5 transgenic mice. Samples were then homogenized in 2 ml of acetone. The homogenate was centrifuged at 5,000 × *g* for 5 min. The pellet was suspended in 2 ml of 0.2 n NaOH, and then incubated overnight at room temperature. Samples were neutralized with 4 n HCl and then treated with DNase I and RNase A (0.04 mg/ml each) (Roche) in 50 mm Tris-HCl, pH 8.0, 10 mm MgCl_2_ for 3 h at 37 °C. Subsequently, the samples were treated with actinase E (0.08 mg/ml) (Kaken Pharmaceutical Co., Ltd., Tokyo, Japan) overnight at 37 °C. Samples were heated to inactivate enzymes and then centrifuged 5,000 × *g* at 4 °C for 10 min. The supernatant was collected, mixed with an equal volume of 50 mm Tris-HCl, pH 7.2, and then applied to a DEAE-Sepharose column for chromatography. The column was washed with 50 mm Tris-HCl, pH 7.2, 0.1 m NaCl. Keratan sulfate bound to the column was eluted with 50 mm Tris-HCl, pH 7.2, 2 m NaCl and then precipitated with ethanol. Keratan sulfate was pre-treated with a mixture of 50 milliunits of *A. ureafaciens* sialidase (Nacalai Tesque, Kyoto, Japan) and 2 microunits of *Streptomyces* sp. 142 α1,3/α1,4 l-fucosidase (Takara Bio Inc., Shiga, Japan) for 2 h at 37 °C. Keratan sulfate was precipitated by ethanol and then digested with 0.5 milliunits of keratanase II (*Bacillus* sp. Ks 36, Seikagaku, Tokyo, Japan) at 37 °C overnight. The oligosaccharide compositions of keratan sulfate were determined by reversed-phase ion-pair chromatography with post-column fluorescent labeling (36).

## RESULTS

### 

#### 

##### Siglec-F Ligands in Blood Are Predominantly Expressed on Eosinophils and Neutrophils

To determine the expression pattern of Siglec-F ligands among leukocyte subsets in peripheral blood, we performed multiparameter flow cytometry using a Siglec-F-Fc fusion protein with the Fc region derived from human IgG_1_. The same construct was used to establish preferential binding of Siglec-F to Gal6S-containing glycans (24). Cells were also stained with Siglec-E-Fc and CD22-Fc to assess the selectivity of Siglec-F-Fc binding. Human IgG did not stain any of the leukocyte populations examined. We took advantage of IL4-GFP reporter (4get) mice to identify eosinophils and basophils, based on their selective expression of GFP (43, 52). We simultaneously stained blood cells with antibodies specific for markers of the major leukocyte lineages; CD3ϵ for T cells, CD19 for B cells, NK1.1 for NK cells, CD49b for basophils and NK cells, Ly-6G for neutrophils, and Ly-6C for classical monocytes.

**FIGURE 1.**
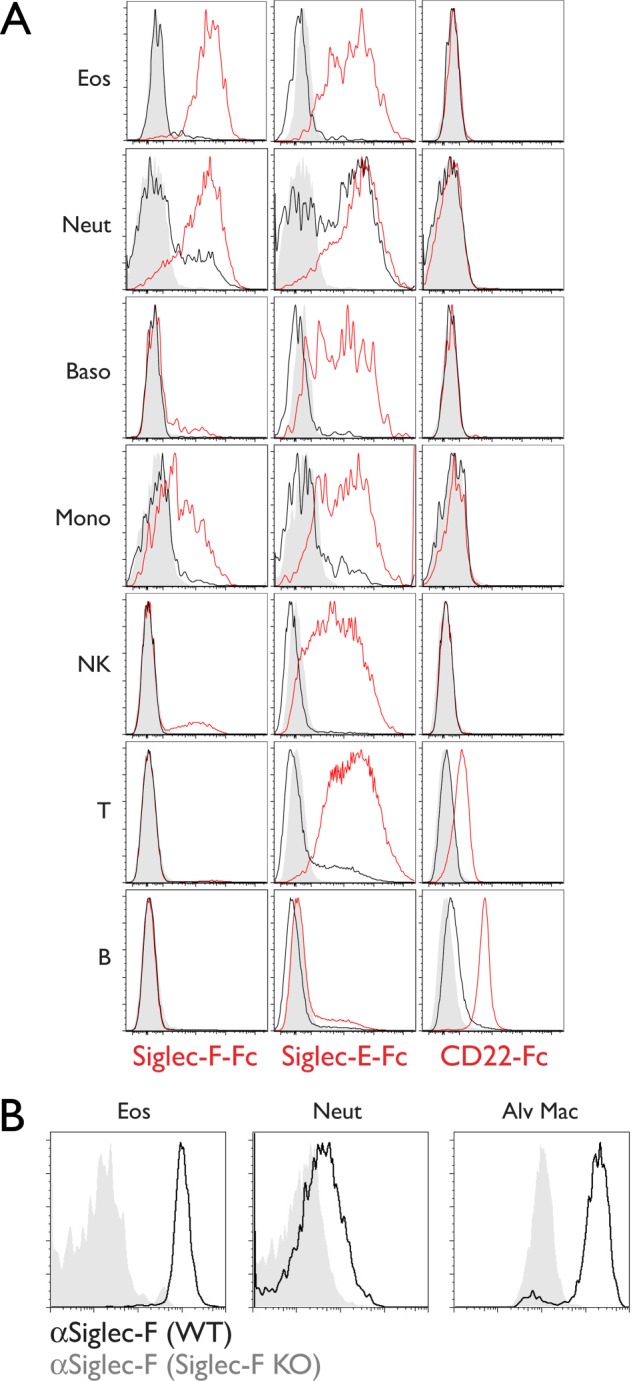
**Siglec-F ligand expression in peripheral blood leukocytes.** *A,* flow cytometry analysis of leukocyte subsets stained with Siglec-F-Fc, Siglec-E-Fc, and CD22-Fc (*red histograms*). Staining after sialidase treatment (*black histograms*) and staining with human IgG (*gray histograms*) are shown. Results are representative of four independent experiments. *B,* flow cytometry analysis of Siglec-F expression on leukocytes from wild type (*black histograms*) or Siglec-F KO mice (*gray histograms*). Results are representative of two independent experiments. *Eos*, eosinophils; *Neut*, neutrophils; *Baso*, basophils; *Mono*, classical monocytes; *NK*, natural killer cells; *T*, T cells; *B*, B cells; *Alv Mac*, alveolar macrophages.

Siglec-F-Fc stained nearly all peripheral blood eosinophils (97 ± 3%, *n* = 4 separate experiments) (Fig. 1*A*). This staining was completely dependent on sialic acid, because sialidase treatment of the cells reduced the signal to background. Staining on neutrophils was of comparable intensity (79 ± 21% of eosinophil MFI). A fraction of neutrophils was resistant to sialidase treatment (20 ± 11%), perhaps indicating the expression of a resistant form of sialic acid (53). Weaker Siglec-F-Fc staining (55 ± 8% of eosinophil MFI) was observed on a population (31 ± 11%) of monocytes, and a similar level of staining (46 ± 10% of eosinophil MFI) was observed on a small population of NK cells (13.9 ± 3.4%). Staining was not detected on basophils, T cells, or B cells. Notably, neutrophils express Siglec-F on their surface, albeit at almost ∼30-fold lower levels than eosinophils (17). We confirmed that this staining was specific, based on its absence in Siglec-F KO mice (Fig. 1*B*). Siglec-E-Fc stained all leukocyte subsets examined except B cells (Fig. 1*A*). CD22-Fc recognized B cells and T cells as previously reported (54), but did not detectably react with eosinophils, neutrophils, basophils, monocytes, or NK cells.

##### Alveolar Macrophages and Type II Alveolar Epithelial Cells Express Siglec-F Ligands

**FIGURE 2.**
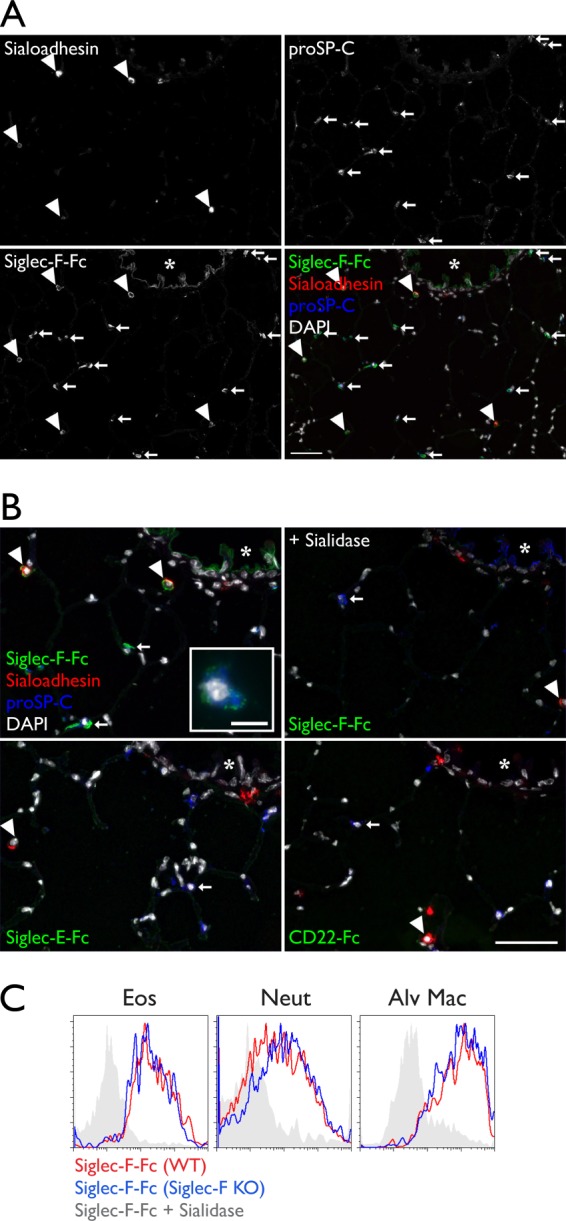
**Siglec-F ligand expression in resident lung cells.** *A,* cryostat-cut sections of lungs from WT mice stained with Siglec-F-Fc (*green*), anti-sialoadhesin (*red*), anti-proSP-C (*blue*), and DAPI (*white*). *Arrowheads* mark alveolar macrophages. *Arrows* mark type II alveolar epithelial cells. The *asterisk* marks airway epithelium. *B,* a high power view of the same section shown in *A*. Siglec-F-Fc staining after sialidase treatment, Siglec-E-Fc staining, and CD22-Fc staining are shown. The *inset* depicts a type II AEC from a different field (*scale bar* 10 μm). Results are representative of four independent experiments. *C,* flow cytometry analysis of Siglec-F-Fc staining of leukocytes from wild type (*red histograms*) or Siglec-F KO (*blue histograms*) mice. Sialidase treatment eliminated staining on cells from both Siglec-F KO (*gray histograms*) and wild type mice (not shown). Results are representative of two independent experiments. *Eos*, eosinophils; *Neut*, neutrophils; *Alv Mac*, alveolar macrophages. *Scale bars* represent 50 μm unless otherwise stated.

Because Siglec-F-Fc is reported to stain epithelial cells in the airways and mononuclear cells in alveoli (17), we sought to determine the identities of the reactive cell types. We stained lung sections with Siglec-F-Fc, and simultaneously stained for sialoadhesin to mark macrophages (55) and pro-surfactant protein C (proSP-C) to mark type II AEC (56) (Fig. 2*A*). We observed Siglec-F-Fc staining in alveolar macrophages. Thus, like eosinophils and neutrophils, these cells express both Siglec-F and Siglec-F ligands. In contrast, Siglec-F-Fc staining was not detected on sialoadhesin^+^ peribronchiolar macrophages (Fig. 2*A*), and this population also lacked Siglec-F expression (data not shown). The pattern of Siglec-F-Fc staining on leukocytes could conceivably be explained by the capture of soluble polyvalent ligands by Siglec-F, which would then be recognized by Siglec-F-Fc. However, Siglec-F-Fc staining of eosinophils, neutrophils, and alveolar macrophages was similar between Siglec-F KO and wild type mice, ruling out this possibility (Fig. 2*C*).

We also found that Siglec-F-Fc stained type-II alveolar epithelial cells (Fig. 2*A*). At high magnification, the reactivity in these cells was punctate and often polarized toward the alveolar space, but it did not co-localize with granules containing proSP-C. We observed Siglec-F-Fc staining of the airway epithelium, as has been reported (17), and we found that it was largely restricted to the luminal surface of the epithelial cells. Staining in the lung was not observed when Siglec-E-Fc or CD22-Fc was used, even though these reagents stained cells in lymph node tissue sections (data not shown). Siglec-F-Fc staining was reduced to the background level when sections were pretreated with sialidase. When we simultaneously stained for Siglec-F ligands, macrophages, and type II AEC, every Siglec-F-Fc^+^ cell was either sialoadhesin^+^, proSP-C^+^, or exhibited a localization consistent with airway epithelium (Fig. 2*B*). Thus, alveolar macrophages, type II epithelial cells, and airway epithelial cells constitute the major classes of Siglec-F ligand-expressing cells in normal lung.

##### Siglec-F Ligands Are Present in Mucin Containing Fractions of BAL Fluid

**FIGURE 3.**
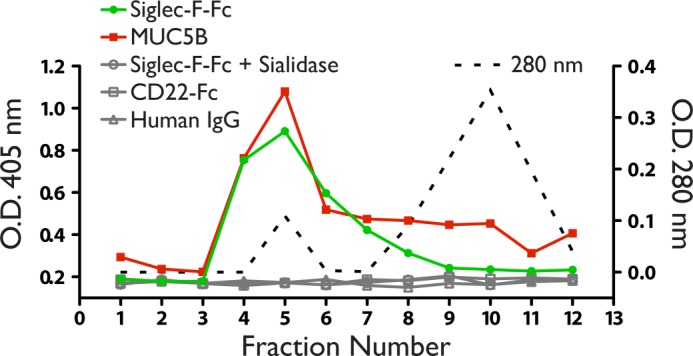
**Siglec-F ligands in BAL fluid.** *A,* fractions of BAL fluid assayed by ELISA using Siglec-F-Fc (*filled circles*), or anti-MUC5B (*filled squares*). Total protein was determined by measuring absorbance at 280 nm (*dotted line*). No signal was observed when wells were reacted with CD22-Fc (*open squares*), human IgG (*open triangles*), or treated with sialidase before incubation with Siglec-F-Fc (*open circles*). Results are representative of two independent experiments.

Mucins are excellent candidates for Siglec ligands due to their abundant sialylated *O-*glycans (57). Additionally, Siglec-F-Fc stains the apical surface of the airway epithelium as well as material in the lumen, where mucins are present (17). To determine whether polymeric secreted mucins could be recognized by Siglec-F, we followed methods to separate high molecular weight mucins from other proteins in airway secretions (48). We concentrated mouse BAL fluid by lyophilization and then solubilized this material in guanidine before fractionating the components by Sepharose CL-2B gel chromatography. The majority of proteins, as monitored by absorbance at 280 nm, eluted in the internal volume of the column (fractions 8–12) (Fig. 3). We then assayed fractions for Siglec ligands and MUC5B by ELISA. MUC5B, which forms complexes of 2–50 million daltons, was enriched in the void volume and the adjacent fractions, consistent with previous findings (48). Siglec-F ligand activity was also highly enriched in these fractions. Siglec-F-Fc reactivity was completely eliminated by sialidase treatment. Neither CD22-Fc nor human IgG was reactive with any fraction.

##### Siglec-F Ligand Expression Increases during Parasitic Worm Infection

Siglec-F ligand expression in airway epithelium and unidentified peribronchiolar mononuclear cells was found to increase during ovalbumin-induced inflammation in mice (17). From our findings above, the accumulation of eosinophils and secreted mucins that accompany allergic lung inflammation could potentially account for this increase. To determine whether Siglec-F ligands increase during another model of lung inflammation, we infected mice with the nematode *N. brasiliensis*. The larvae of this parasitic worm travel from the subcutaneous injection site through the vasculature to the lung. They then migrate through the alveoli and ascend the airways before entering the gastrointestinal tract (49). As a result of parasite migration through the lung, the airway epithelium thickens and there is increased mucus production and eosinophil accumulation (58, 59).

**FIGURE 4.**
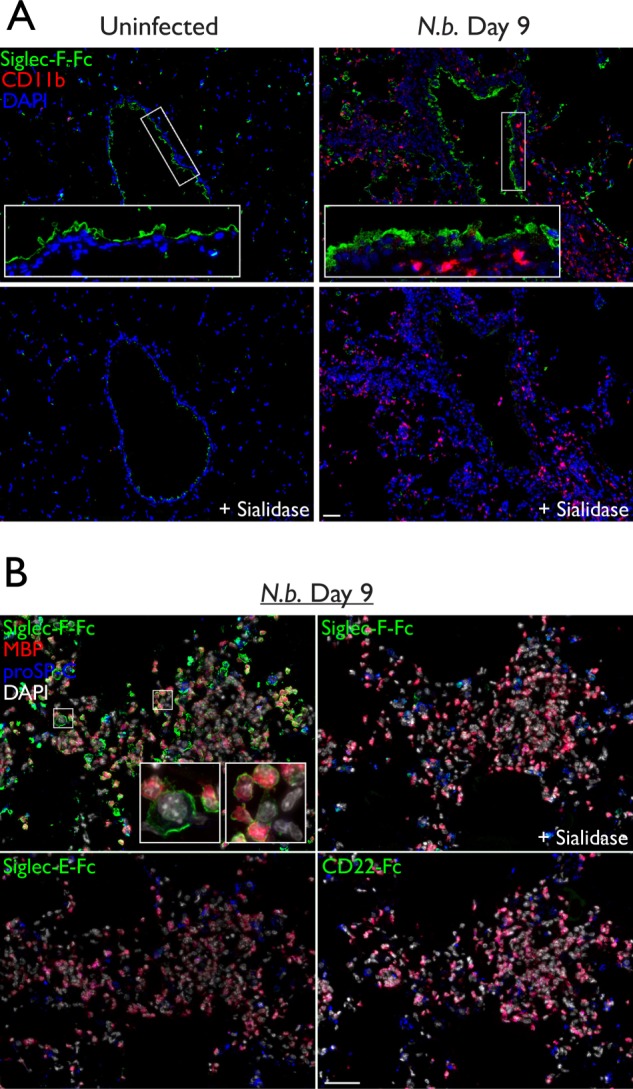
**Siglec-F ligand expression during *N. brasiliensis* infection.** *A,* cryostat-cut serial sections of lungs from *N. brasiliensis* infected or uninfected mice. Sections were treated with sialidase or buffer alone, then stained with Siglec-F-Fc (*green*), anti-CD11b (*red*), and DAPI (*blue*). *B,* high power images of the same lung sections from *A*, treated with sialidase or buffer alone, then stained with Siglec-F-Fc, Siglec-E-Fc, or CD22-Fc (*green*); anti-eMBP (*red*); anti-proSP-C (*blue*); and DAPI (*white*). *Scale bar* represents 50 μm. Results are representative of two independent experiments.

Compared with uninfected lung, the airway epithelium showed increased Siglec-F-Fc reactivity, which was present in the cytoplasm of epithelial cells as well as on the apical surface (Fig. 4*A*). There was also an increase in ligands in the alveolar spaces where CD11b^+^ myeloid cells had accumulated. To identify eosinophils, we used an antibody specific for eosinophil major basic protein (eMBP), a component of the primary granules in these cells (1). As expected, many of the infiltrating cells were eMBP^+^ eosinophils, and these cells stained with Siglec-F-Fc (Fig. 4*B*).

##### Analysis of Sulfated Glycans in Eosinophils and BAL Fluid

**FIGURE 5.**
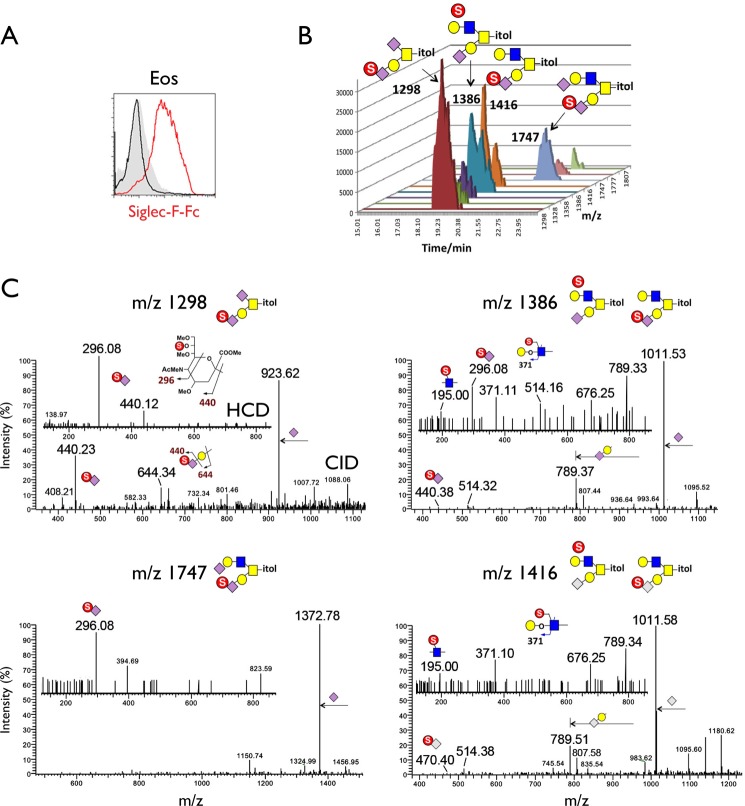
**MS analysis of sulfated glycans in eosinophils.** *A*, flow cytometry analysis of IL-5 transgenic eosinophils stained with Siglec-F-Fc (*red histogram*). Staining after sialidase treatment (*black histogram*) and staining with human IgG (*gray histogram*) is shown. Results are representative of three independent experiments. *B,* extracted ion chromatograms of the major sulfated *O-*glycans from IL-5 transgenic eosinophils as detected by nanoLC-MS/MS analysis. The *m*/*z* values for the [M − H]^−^ molecular ions afforded by the mono-sulfated permethylated *O-*glycans were annotated along with the assigned structures based on interpretation of the HCD and CID MS/MS data. The relative peak heights are indicative of the relative abundance of each of the sulfated, sialylated core 1 and core 2 *O-*glycans. *C,* low mass regions of the negative ion mode nanoESI HCD and CID MS/MS spectra of mono-sulfated di-sialylated (*left*) and mono-sulfated mono-sialylated (*right*) structures. Assignment of the major peaks for all spectra is annotated using the standard schematic symbols. *Eos*, eosinophils.

As reviewed above, Siglec-F exhibits striking specificity for glycans containing Gal6S. However, this modification has not been identified on eosinophils or mouse airway mucins, which are implicated as sources of biological ligands for Siglec-F. We carried out a sulfoglycomic analysis to determine whether Gal6S was present on the *N*- or *O-*glycans from these sources. This determination was based on diagnostic fragment ions afforded by nanoLC-MS/MS analysis of permethylated sulfated glycans in negative ion mode, as described previously (36). We first collected large numbers of peripheral blood eosinophils (120 × 10^6^ cells, 81% eosinophils, 9% neutrophils) from mice constitutively expressing the eosinophil survival factor IL-5 under the control of the mouse CD3δ regulatory regions (44). We verified that eosinophils from these mice exhibited Siglec-F-Fc staining comparable with that of wild type eosinophils (Fig. 5*A*). From this sample, the non-sulfated *N*-glycans were found to comprise the usual range of high mannose and complex type structures, with the latter mostly terminating in Neu5Ac/Neu5Gc or LacNAc sequences (data not shown), very similar to the profile mapping undertaken previously by the Consortium for Functional Glycomics. Screening for structures corresponding to sulfated *N*-glycans did not afford any signals above the noise level, except for those assigned as Man-6-phosphate-carrying high mannose structures. Such structures are commonly found by this analytical approach (60) when sulfated *N*-glycans are scarce. LC-MS/MS analysis of the sulfated *O-*glycan fraction, on the other hand, afforded several weak signals (Fig. 5*B*) that could be assigned as the mono-sulfated counterparts of the few mono- and di-sialylated simple core 1 (Galβ1→3GalNAc) and core 2 (Galβ1→3(GlcNAcβ1→6)GalNAc) structures identified in the non-sulfated fraction. Importantly, when we selected these sulfated structures for MS/MS analysis, the diagnostic ion at *m*/*z* 167 that would implicate 6-*O*-sulfate on either a terminal Gal or an α2,3-sialylated Gal was not detected above the noise level (Fig. 5*C*). Instead, we observed fragment ions indicative of Gal→(6S)GlcNAc (*m*/*z* 195 and 371) in the mono-Neu5Ac/Neu5Gc-sialylated core 2 structures (*m*/*z* 1386, 1416). Unexpectedly, our MS/MS data showed that the most abundant sulfated *O-*glycans appeared to carry the sulfate on the glycerol side chain of Neu5Ac (*m*/*z* 296, 440) in both core 1 and core 2 structures.

**FIGURE 6.**
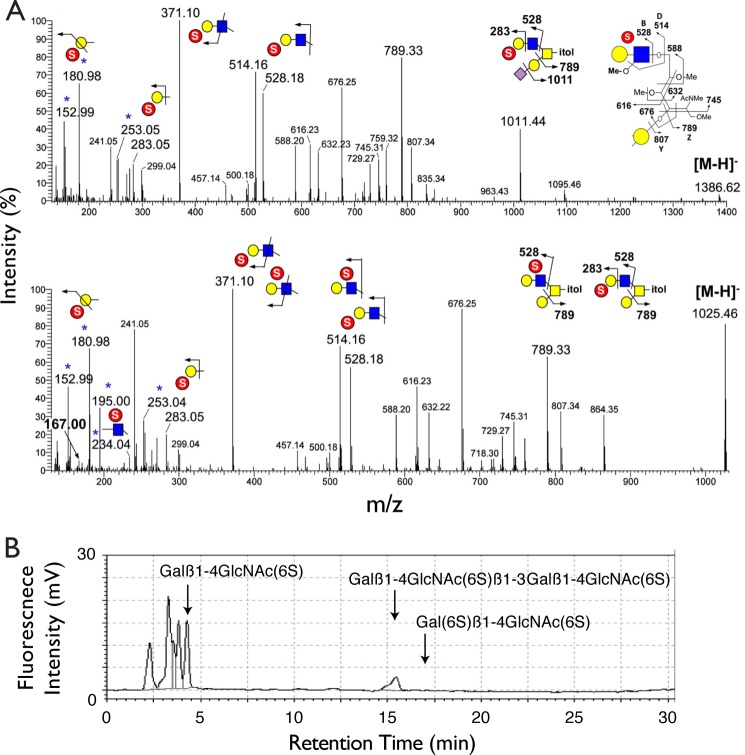
**Analysis of sulfated glycans in BAL fluid and lung KS.** *A*, representative negative ion mode nanoESI HCD MS/MS spectra of mono-sulfated mono-sialylated (*top*) and mono-sulfated non-sialylated (*bottom*) *O-*glycan structures found in BAL fluid from wild type mice. Identification of terminal Gal3S and internal GlcNAc6S was based on previously established diagnostic ions. A weak signal at *m*/*z* 167 (*bold*) may indicate the presence of Gal6S in the non-sialylated structures. Additional clusters of fragment ions around *m*/*z* 500–800 are assigned as shown, which are mostly fragment ions resulting from cleavage along the GalNAcitol and consistent with the sulfated LacNAc being extended from the 6-arm. *B,* reversed-phase ion-pair chromatography analysis of KS from lungs of wild type mice. Standard substances were eluted at the peak positions indicated by *arrows*. The fragment (6S)Galβ1→4(6S)GlcNAc was not detected in the sample.

The diagnostic fragment ion indicative of Gal6S was likewise not detected in the MS/MS spectra of sulfated, sialylated core 2 *O-*glycan structures derived from total BAL fluid of wild type mice (Fig. 6*A*). These sulfated mono-sialylated structures were found to carry primarily a Gal3S (*m*/*z* 153 and 181) or GlcNAc6S (*m*/*z* 195, 234) on sulfated, non-sialylated LacNAc (*m*/*z* 371). Only the non-sialylated core 2 structures (*m*/*z* 1025) afforded a minimal signal at *m*/*z* 167, which could indicate the presence of a minor amount of Gal6S.

Extended, polysulfated LacNAc chains, such as those present in keratan sulfate (KS), often contained Gal6S (61). Because these structures can be capped with sialic acid (62), they have the potential to serve as Siglec-F ligands. However, analysis of such chains was not achievable by our mass spectrometry methods. Therefore, we used HPLC to analyze KS chains for the presence of Gal6S in whole mouse lung tissue. This methodology has previously been employed by us to reveal both Gal6S and GlcNAc6S on KS from adult mouse eyes (36). Although GlcNAc6S was abundant on KS in lung samples, we did not detect Gal6S (Fig. 6*B*). We also analyzed a large sample of mouse eosinophils, but found no evidence for the presence of KS (data not shown).

##### Gal 6-O-sulfotransferases Are Not Required for the Generation of Siglec-F Ligands

The lack of detectable Gal6S in sialylated glycans from eosinophils and BAL fluid suggested that this modification was not required for Siglec-F ligand recognition. However, it was conceivable that Gal6S below the limit of detection still contributed to Siglec-F-Fc staining in these samples. Therefore, we sought to determine whether the two known Gal6STs were required for the generation of endogenous Siglec-F ligands. We crossed KSGal6ST KO mice with C6ST-1 KO mice to generate KSGal6ST/C6ST-1 DKO mice. Both strains of mice have been verified for gene deletion and absence of enzyme function (36, 42).

**FIGURE 7.**
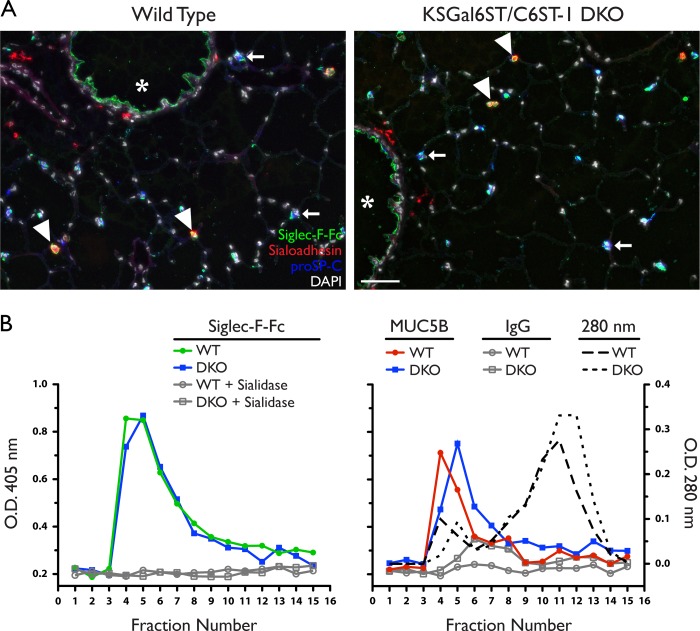
**Siglec-F ligand expression in lungs from KSGal6ST/C6ST-1 DKO mice.** *A,* cryostat-cut sections of lungs from WT or DKO mice stained with Siglec-F-Fc (*green*), anti-sialoadhesin (*red*), anti-proSP-C (*blue*), and DAPI (*white*). *Scale bar* represents 50 μm. Results are representative of two independent experiments. *B,* Siglec-F-Fc reactivity (*left*) in BAL fluid fractions from WT (*filled circles*) or DKO mice (*filled squares*), assayed by ELISA. Results are representative of two independent experiments. The signal was eliminated by sialidase treatment (*open circles*, *open squares*). Anti-MUC5B reactivity (*right*) in BAL fluid fractions from WT (*filled circles*) and DKO mice (*filled squares*) was assayed by ELISA. Isotype control signal was minimal (*open circles*, *open squares*). Total protein was determined by measuring absorbance at 280 nm for wild type (*dotted line*) and DKO mice (*dashed line*). WT, wild type; DKO, KSGal6ST/C6ST-1 double knock-out.

We first stained lung tissue sections from DKO mice with Siglec-F-Fc and found that staining was unchanged in alveolar macrophages, type II AECs, and airway epithelial cells in the absence of Gal6STs (Fig. 7*A*). When we titrated the concentration of Siglec-F-Fc to the limit of detection, there was no difference in staining between wild type and DKO mice (data not shown). We also analyzed Siglec-F reactivity in fractionated BAL fluid from DKO mice (Fig. 7*B*). Siglec-F-Fc binding in the void volume fractions was indistinguishable between wild type and DKO mice, consistent with the epithelial staining we observed in tissue sections. BAL fluid from wild type and DKO mice contained similar amounts of MUC5B in these fractions.

**FIGURE 8.**
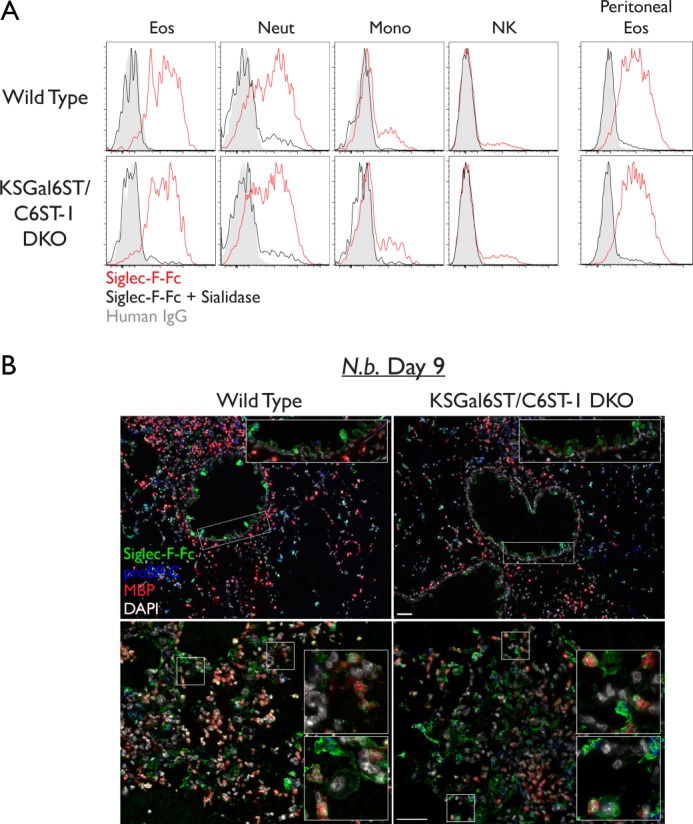
**Siglec-F ligand expression in leukocytes from KSGal6ST/C6ST-1 DKO mice.** *A,* flow cytometry analysis of leukocyte subsets stained with Siglec-F-Fc (*red histograms*). Staining after sialidase treatment (*black histograms*) and staining with human IgG (*gray histograms*) are shown. *Scale bars* represent 50 μm. Results are representative of two independent experiments. *B,* cryostat-cut sections of lungs from *N. brasiliensis*-infected mice. Sections were stained with Siglec-F-Fc (*green*), anti-eMBP (*red*), anti-proSP-C (*blue*), and DAPI (*white*). Low power (*top*) and high power (*bottom*) fields are shown. *Eos*, eosinophils; *Neut*, neutrophils; *Mono*, classical monocytes; *NK*, natural killer cells.

Next, we performed Siglec-F-Fc staining on peripheral blood leukocytes from wild type and DKO mice. We found that Siglec-F-Fc staining was unchanged in eosinophils, neutrophils, monocytes, and NK cells from DKO mice compared with that in wild type mice (Fig. 8*A*). We also found that Siglec-F-Fc staining was similar between wild type and DKO eosinophils obtained during thioglycolate-induced peritonitis. Finally, we stained lung sections from DKO mice with Siglec-F-Fc during *N. brasiliensis* infection and found epithelial cell and eosinophil staining comparable to that observed in infected wild type mice (Fig. 8*B*).

## DISCUSSION

Here, we demonstrate that the cell types previously known to express Siglec-F, namely eosinophils, neutrophils, and alveolar macrophages (15–18), correspondingly express ligands for this receptor on their surface. This is consistent with results that were published while this manuscript was under review (27). We verify that all of these ligands require sialylation. Furthermore, these ligands cannot be explained by Siglec-F-mediated presentation of a soluble polyvalent ligand or by Siglec-F itself functioning as a ligand, because we did not detect changes in ligand expression in Siglec-F KO mice. The ligands detected on eosinophils may correspond to previously identified cis-ligands, which prevent the binding of a 6′-sulfo-sLex-based probe to the eosinophil cell surface (24). It remains to be seen whether the ligands on neutrophils and alveolar macrophages also serve as cis-ligands for Siglec-F. As has been shown for CD22, cis-ligands can act as trans-ligands when cells come into contact with one another, and these contacts have functional significance in T cell-B cell interactions (63). Thus, ligand expression on eosinophils, neutrophils, and alveolar macrophages may indicate important bi-directional trans-interactions between these cell types.

We have also demonstrated that Siglec-F ligands are present in high-molecular weight fractions of mouse BAL fluid. These fractions contain polymeric secreted mucins in humans (48), and indeed we verified the co-elution of MUC5B. Mucins are highly sialylated glycoproteins that can be modified with hundreds of *O-*glycans per molecule (57), and they have been shown to serve as ligands for carbohydrate-binding proteins. For example, mucins constitute functional ligands for L-selectin (64, 65), CLEC-2 (66), and galectins (67–69). Additionally, mucins have also been implicated as ligands for a number of Siglecs, including sialoadhesin, CD22, CD33, MAG, and Siglec-9 (10–12, 70–72). The BAL fluid ligands we characterized are likely to require expression of ST3Gal3, because Siglec-F-Fc staining of airway epithelium is lost in mice deficient in this enzyme (26, 27). We detected Siglec-F ligands on the apical membranes of airway epithelial cells but rarely in the cytoplasm, which is consistent with the localization of MUC5B in normal airways (73). Additionally, there was a marked increase in ligands during worm infection, some of which were associated with the apical cytoplasm and cell surface of epithelial cells where secreted mucins are abundant (58). However, we have not ruled out the possibility that integral membrane proteins (mucins or otherwise) also serve as ligands on airway epithelial cells. Further studies with mice deficient in specific mucin genes may allow identification of the protein scaffolds for ligands in BAL fluid. We also found punctate Siglec-F-Fc staining in type II AECs. Like mucin-producing cells, type II AECs are highly secretory, releasing surfactant from lamellar granules into the alveoli (74). It is possible that these cells secrete ligands into the air spaces, which could account for the weak Siglec-F-Fc signal in the low molecular weight fractions of BAL fluid.

A dramatic increase in Siglec-F ligand expression has been observed during ovalbumin-induced allergic lung inflammation (17). We have extended this observation to inflammation that develops in response to the parasitic nematode, *N. brasiliensis*. Eosinophil accumulation and increased mucus production are features of both of these models (75). Given that we detect ligands on eosinophils and in mucin-containing fractions of BAL fluid, these sources likely contribute to the increased Siglec-F-Fc staining seen during allergic lung inflammation. Eosinophils and neutrophils are known to migrate across the airway and alveolar epithelium (76), and alveolar macrophages reside in the alveolar lumen. As reviewed above, previous studies have implicated Siglec-F in the regulation of eosinophil survival (17). Thus, ligands secreted into the airways by epithelial cells, and into alveoli by type II AECs, could potentially function to regulate the activation or survival of all three Siglec-F-expressing leukocytes in the luminal spaces of the lung.

There is also potential for Siglec-F-mediated interactions, both homotypic and heterotypic, between eosinophils, neutrophils, and alveolar macrophages during inflammation. We observed many examples of apparent cell-cell contact between these cell types in the inflamed lung. Alveolar macrophages are known to engage in phagocytosis of granulocytes (77). An intriguing possibility is that Siglec-F ligation by cell surface ligands limits activation of both the granulocyte and the alveolar macrophage during this process. Siglec-F is an endocytic receptor (78), and may also be directly involved in the uptake of these cells. Siglec-F-deficient mice exhibit general signs of enhanced allergic lung inflammation, such as increased airway smooth muscle thickness and systemic increases in eosinophil numbers (17, 22). Investigation of this phenotype has focused on effects intrinsic to eosinophils. However, future studies should also include the potential functions of Siglec-F on alveolar macrophages and neutrophils.

Screening with glycan arrays has implicated Gal6S as a recognition element for Siglec-F (24). However, we found that the two known Gal6STs are not required for the generation of Siglec-F ligands. It is possible that another Gal6ST exists; however, we consider this unlikely due to the high degree of sequence similarity among members of the GST family (31, 79). Additionally, we have recently shown that KSGal6ST alone is responsible for generating a variety of Gal6S-containing glycans in the eye and lymph node (36). Finally, Gal6S was not detected in sialylated glycans from either eosinophils or BAL fluid, although we detected other sulfated monosaccharides in these samples. The possible presence of Gal6S on non-sialylated core 2 structures, but not their sialylated counterparts in BAL fluid could indicate competition between α2,3-sialyltransferases and Gal6STs. It should be noted that we were readily able to detect Gal6S in glycan standards and lymph node *O-*glycans using the same analytic methods (36). Therefore, we conclude that Gal6S is unlikely to be required for the generation of any of the classes of Siglec-F ligands investigated here.

We did not detect sulfated *N*-glycans in eosinophils, whereas sulfated *O-*glycans were clearly present. This likely reflects a paucity of *N*-glycan sulfation in this cell type, because non-sulfated and Man-6-phosphate-containing *N*-glycans were abundant. Our characterization of sulfated glycans is partial and importantly, does not include glycolipids. However, we are not aware of evidence for the presence of Gal6S on glycolipids in mammals. The ligands in high molecular weight fractions of BAL fluid are most likely glycoproteins. Glycan array studies have revealed that several other Siglecs and C-type lectins bind to glycans containing Gal6S, including Siglec-E, Siglec-8, Siglec-7, Siglec-5, and Langerin (29, 37, 46). In view of the present findings regarding Siglec-F specificity, caution is advised in drawing conclusions about the involvement of Gal6S in the biological ligands for these receptors.

Siglec-F and Siglec-8 are not orthologous genes. Siglec-F contains one more C2-set Ig domain than Siglec-8, and is closest in sequence homology to Siglec-5 (80). Additionally, Siglec-F and Siglec-8 have somewhat divergent expression patterns. Siglec-8 is not expressed on human neutrophils or alveolar macrophages, whereas Siglec-F is not expressed on mouse mast cells (81). Moreover, the expression of endogenous ligands for these two receptors appears to be different, because Siglec-8-Fc does not stain human airway epithelium or eosinophils (30). Both receptors recognize 6′-sulfo-sLex and 6′-sulfo-3′sLN, although Siglec-F binds several other structures on glycan arrays. We also provide evidence here that Gal6S is not required for recognition of endogenous ligands for Siglec-F. These findings engender skepticism that native Siglec-8 ligands involve Gal6S, but this question remains to be addressed directly.

The selectivity of Siglec-F-Fc staining among leukocytes is likely based on the presence of a highly specific terminal sialylated glycan. The structural definition of this glycan remains an important area for future work. Sialic acid itself can be modified with hydroxyl, methyl, acetyl, phosphate, and sulfate groups to generate over 50 variations (82). Interestingly, sulfo-Neu5Ac with the sulfate moiety on the 3-carbon glycerol side chain was the most prevalent sulfated monosaccharide we detected in glycans from enriched mouse eosinophils. Sulfo-sialic acid has been detected in sea urchin, and various mouse and human tissues (83–86). However, the functions of this modification and the enzymes responsible for its synthesis are not known. It will be of great interest to examine eosinophils, alveolar macrophages, and neutrophils to determine whether the presence of particular sialylated glycans, including those containing sulfo-sialic acid, can be correlated with Siglec-F ligand expression.

## Footnotes

2 The abbreviations used are:

Siglec: sialic acid-binding immunoglobulin-like lectin
C6ST-1: chondroitin 6-*O*-sulfotransferase-1
Gal6S: galactose 6-*O*-sulfate
Gal6ST: galactose 6-*O*-sulfotransferase
KSGal6ST: keratan sulfate galactose 6-*O*-sulfotransferase
proSP-C: pro-surfactant protein C
Gal: galactose
Glc: glucose
GlcNAc: *N*-acetylglucosamine
GST: GlcNAc/Gal/GalNAc-6-*O*-sulfotransferase
Fuc: fucose
Sia: sialic acid
Neu5Gc: *N*-glycolylneuraminic acid
LacNAc: *N*-acetyllactosamine
KO: knock-out
APC: allophycocumarin
Cy3: indocarbocyanine
LC: liquid chromatography
MALDI: matrix-assisted laser desorption/ionization
ESI: electrospray ionization
AEC: alveolar epithelial cell
BAL: bronchoalveolar lavage
CID: collision-induced dissociation
HCD: higher energy C-trap dissociation
eMBP: eosinophil major basic protein
KS: keratan sulfate
DKO: double knock-out
PE: phosphatidylethanolamine
MFI: mean fluorescence intensity
PAPS: 3′-phosphoadenosine 5′-phosphosulfate.
